# Using Oxford Nanopore Sequencing to Investigate Potential Plasmid Transmission of the blaNDM-5 Gene

**DOI:** 10.1017/ash.2025.347

**Published:** 2025-09-24

**Authors:** Chin-Ting Wu, Guy Handley, William Shropshire, Sherry Cantu, Jane Powell, Micah Bhatti, Samuel Shelburne, Amy Spallone

**Affiliations:** 1 The University of Texas MD Anderson Cancer Center; 2 MD Anderson Cancer Center; 3 University of Texas MD Anderson Cancer Center

## Abstract

**Background:** The transmission of plasmids carrying antimicrobial resistance (AMR) genes between patients poses a significant global health challenge. The New Delhi metallo-β-lactamase (NDM) carbapenemase is associated with high mortality and limited therapeutics. Traditional Infection Control surveillance for intra-institutional organisms carrying AMR genes relies on retrospective clinical and epidemiologic review. Whole-genome sequencing (WGS) has been utilized to detect outbreaks, including those not clinically apparent within healthcare settings. In this study, we sought to identify related NDM-carrying Escherichia coli using WGS. **Method:** NDM-carrying E. coli isolates in clinical cultures were identified between November 2023 and October 2024. Bacterial genomic DNA was extracted directly from clinical microbiology laboratory plates. Sequencing was performed using the Oxford Nanopore Technologies MinION platform with an R10.4.1 flow cell. Assembled FASTA files from the in-house Flyest pipeline were analyzed with AMRFinderPlus to detect AMR genes. Plasmid replicons were identified using staramr. Horizontal plasmid transfer annotation was conducted using Roary, which incorporates Prokka, and pairwise single nucleotide polymorphism distances were assessed with snps-dist. **Results:** Nine E. coli strains producing blaNDM-5 were identified from eight patients. Two isolates from one patient showed high genetic similarity, indicating they were likely the same strain. The identified sequence types (STs) were ST361 (4 strains), ST167 (3 strains), ST205 (1 strain), and ST405 (1 strain). Four strains harbored a hybrid IncFIA/IncFIB(AP001918) plasmid, demonstrating 99.8%–99.9% nucleotide similarity. This plasmid carried blaNDM-5, blaCTX-M-15, blaOXA-1, and efflux pump-related genes, contributing to multidrug resistance. Despite the high plasmid similarity, no obvious epidemiological links, such as overlapping patient admissions or common procedures, were identified among the four patients carrying this hybrid plasmid. **Conclusion:** WGS presents a novel modality to identify highly genetically related strains of organisms within a healthcare institution that may reflect silent outbreaks or AMR dissemination not detected by conventional Infection Control methods.

